# Effects of different combinations of mechanical loading intensity, duration, and frequency on the articular cartilage in mice

**DOI:** 10.1007/s11033-024-09762-5

**Published:** 2024-07-29

**Authors:** Yoshio Wakimoto, Yasushi Miura, Shota Inoue, Masato Nomura, Hideki Moriyama

**Affiliations:** Department of Rehabilitation Science, Kobe University Graduate School of Health Sciences, Tomogaoka 7-10-2, Suma-ku, 654-0142 Kobe, Hyogo Japan

**Keywords:** Mechanical loading, Cyclic tensile strain, Treadmill exercise, Knee cartilage metabolism, Knee cartilage homeostasis

## Abstract

**Background:**

Understanding how healthy articular cartilage responds to mechanical loading is critical. Moderate mechanical loading has positive effects on the cartilage, such as maintaining cartilage homeostasis. The degree of mechanical loading is determined by a combination of intensity, frequency, and duration; however, the best combination of these parameters for knee cartilage remains unclear. This study aimed to determine which combination of intensity, frequency, and duration provides the best mechanical loading on healthy knee articular cartilage in vitro and in vivo.

**Methods and results:**

In this study, 33 male mice were used. Chondrocytes isolated from mouse knee joints were subjected to different cyclic tensile strains (CTSs) and assessed by measuring the expression of cartilage matrix-related genes. Furthermore, the histological characteristics of mouse tibial cartilages were quantified using different treadmill exercises. Chondrocytes and mice were divided into the control group and eight intervention groups: high-intensity, high-frequency, and long-duration; high-intensity, high-frequency, and short-duration; high-intensity, low-frequency, and long-duration; high-intensity, low-frequency, and short-duration; low-intensity, high-frequency, and long-duration; low-intensity, high-frequency, and short-duration; low-intensity, low-frequency, and long-duration; low-intensity, low-frequency, and short-duration. In low-intensity CTSs, chondrocytes showed anabolic responses by altering the mRNA expression of COL2A1 in short durations and SOX9 in long durations. Furthermore, low-intensity, low-frequency, and long-duration treadmill exercises minimized chondrocyte hypertrophy and enhanced aggrecan synthesis in tibial cartilages.

**Conclusion:**

Low-intensity, low-frequency, and long-duration mechanical loading is the best combination for healthy knee cartilage to maintain homeostasis and activate anabolic responses. Our findings provide a significant scientific basis for exercise and lifestyle instructions.

**Supplementary Information:**

The online version contains supplementary material available at 10.1007/s11033-024-09762-5.

## Introduction

Knee joints bear a majority of the body weight; thus, the articular cartilage of the knee joints is particularly more likely to degenerate [1]. Because chondrocyte metabolism is low and intact cartilage has no blood supply [2], natural cartilage repair is limited. Mechanical loading is an essential factor for regulating cartilage homeostasis [3]; therefore, understanding how healthy articular cartilage responds to mechanical loading is critical for maintaining healthy cartilages.

Moderate mechanical loading has positive effects on increasing the anabolic responses of the cartilage matrix [3], whereas excessive mechanical loading induces catabolic responses [4, 5]. A combination of intensity, frequency, and duration determines the degree of mechanical loading [6]. The effects of these parameters on cartilage response and health have been extensively examined in vitro and in vivo [3, 4, 7–9].

Cyclic tensile strain (CTS) can be applied to chondrocytes in various strain intensities, frequencies, and durations [10]. A review showed that high-intensity, high-frequency, and long-duration CTS increases catabolic responses, and CTS of 3–10% intensity, 0.17–0.5 Hz, and 2–12 h may induce anabolic responses in healthy chondrocytes [8]. However, direct comparison is difficult because different experimental conditions (e.g., animal species, age, and cell type) were used.

Running or walking is one of the most common weight-bearing activities and can simulate long-term mechanical loading on weight-bearing joints [11]. Moderate running increases cartilage thickness and promotes cartilage matrix synthesis and cartilage protection in a healthy animal model [12, 13]. Conversely, high-intensity and long-duration or long-distance running accelerates cartilage matrix degradation and cartilage thinning [3, 9, 11]. In contrast, some studies have reported that the thickness and matrix of healthy cartilage do not change or conversely increase with excessive running [3, 12, 14–17].

Taken together, there is no consensus on the optimal combination of intensity, frequency, and duration for eliciting preferred responses in healthy knee articular cartilage [18, 19]. Therefore, this study aimed to determine which combination of intensity, frequency, and duration provides the best mechanical loading on healthy knee articular cartilages.

## Materials and methods

### Experimental animals and animal care

In this study, 33 male C57BL/6J mice (7 weeks old, with a mean body weight of 19–24 g) purchased from Japan SLC (Shizuoka, Japan) were used. The animals were housed in standard cages (3 mice/cage) under a 12-h dark/light cycle at a constant temperature of 22 °C ± 1 °C and allowed free access to water and standard foods. Six mice were used for CTS, and 27 mice were used for treadmill exercises.

### Isolation and culture of chondrocytes

The mice were euthanized by exsanguination under anesthesia, and primary chondrocytes were isolated from the femoral condyles and tibial plateau according to a previous study [20]. After rinsing the cartilage pieces (excluding the subchondral bone that appears brown) with phosphate-buffered saline, chondrocytes were isolated from the cartilage using 0.4% collagenase (034-22363; FUJIFILM Wako Pure Chemical Co., Osaka, Japan) overnight at 37 °C. These chondrocytes were seeded in a 35-mm cell culture dish (353,801; BD Falcon, Tokyo, Japan). They were then cultured in Dulbecco’s Modified Eagle Medium–Ham’s F12 medium (DMEN/HAM’S F-12; 042-30555; FUJIFILM Wako Pure Chemical Co., Osaka, Japan) supplemented with 10% fetal bovine serum (Gibco 12,483,020; Thermo-Fisher Scientific, Inc., MA, USA), 50 units/mL penicillin, and 50 µg/mL streptomycin (168-23191; FUJIFILM Wako Pure Chemical Co., Osaka, Japan) in an incubator maintained at 37 °C with 5% CO_2_. The medium was changed every 3 days.

At up to 80–90% confluency, the chondrocytes were harvested with a trypsin–ethylenediaminetetraacetic acid solution (209-16941; FUJIFILM Wako Pure Chemical Co., Osaka, Japan) for passage. In passage 1, the chondrocytes were seeded at 2.5 × 10^5^ cells in 35-mm culture dishes (353,001; BD Falcon, Tokyo, Japan). Similarly, the chondrocytes were seeded at a 10-cm^2^ chamber (STB-CH-10; Strex Inc., Osaka, Japan) coated with type I collagen (IPC-50; AteloCell, Koken, Tokyo, Japan) in passage 2. Chondrocyte cultures were continued by changing the medium every 3 days.

### Exposure of chondrocytes to CTS and RNA extraction

After the chondrocytes of passage 2 reached 80–90% confluency (approximately 2 weeks after seeding of passage 2), CTS was applied to the chondrocytes using the Strex device (STB-140; Strex Inc., Osaka, Japan). The control group was cultured under the same conditions as the other CTS intervention groups in passage 1 and passage 2. The chondrocytes in the control group were cultured on coated stretch chambers under the same conditions as the other intervention groups in passage 2, and total RNA were extracted from the chondrocytes without CTS intervention when they were 80–90% confluent. In the chondrocytes of several species, including mice, chondrocytes of passage 2 maintain their shape and phenotype. Based on the morphological findings in previous reports [21–23], we confirmed that the chondrocytes of passage 2 exhibit a polygonal shape characteristic of chondrocytes, rather than the elongated fibroblastic shape that reveals dedifferentiated cells. CTS with a sinusoidal waveform was applied to chondrocytes, which were divided into the following nine groups (*n* = 4 chambers): control (no intervention); high-intensity, high-frequency (1.0 Hz), and long-duration (24 h) (HHL); high-intensity (15%), high-frequency, and short-duration (12 h) (HHS); high-intensity, low-frequency (0.5 Hz), and long-duration (HLL); high-intensity, low-frequency, and short-duration (HLS); low-intensity (8%), high-frequency, and long-duration (LHL); low-intensity, high-frequency, and short-duration (LHS); low-intensity, low-frequency, and long-duration (LLL); and low-intensity, low-frequency, and short-duration (LLS) (Table 1). The intensity, duration, and frequency of this CTS protocol were determined based on a previous review [8]. The 12-h time point is considered to be the approximate time point at which the response of chondrocytes to CTS switches from anabolism to catabolism [8]. Immediately after CTS, total RNA was extracted from chondrocytes using ISOSPIN Cell & Tissue RNA (314–08211; NIPPON GENE CO., LTD, Tokyo, Japan), according to the manufacturer’s instructions. The purity and concentration of the extracted total RNA were measured using a BioPhotometer D30 (Eppendorf, Hamburg, Germany).

**Table 1:** Groups and stretch protocols for cyclic tensile stretch of articular chondrocytes

Groups	Intensity (%)	Frequency (Hz)	Duration (hours)
Control (no intervention)	–	–	–
High-intensity, high-frequency, and long-duration (HHL)	15	1.0	24
High-intensity, high-frequency, and short-duration (HHS)			12
High-intensity, low-frequency, and long-duration (HLL)		0.5	24
High-intensity, low-frequency, and short-duration (HLS)			12
Low-intensity, high-frequency, and long-duration (LHL)	8	1.0	24
Low-intensity, high-frequency, and short-duration (LHS)			12
Low-intensity, low-frequency, and long-duration (LLL)		0.5	24
Low-intensity, low-frequency, and short-duration (LLS)			12

### Analysis of gene expression in chondrocytes using quantitative real-time polymerase chain reaction (qRT-PCR)

Reverse transcription and qRT-PCR were performed using the StepOne Real-Time PCR system (Thermo-Fisher Scientific Inc., MA, USA) with the TaqMan™ Fast Virus 1-Step Master Mix (Thermo-Fisher Scientific Inc., MA, USA) and Gene Expression Assays (Applied Biosystems, CA, USA) for collagen type II alpha1 mRNA (*COL2A1*; Mm01309565_m1), aggrecan mRNA (*ACAN*; Mm00545794_m1), sex-determining region Y-box 9 (SOX9) mRNA (*SOX9*; Mm00448840_m1), a disintegrin-like and metallopeptidase with thrombospondin type 1 motif 5 (ADAMTS5) mRNA (*ADAMTS5*; Mm00478620_m1), matrix metallopeptidase 13 (MMP13) mRNA (*Mmp13*; Mm00439491_m1), and 18 S ribosomal(18s) mRNA (*18s*: Mm03928990_g1). Their expression levels were analyzed using the 2^−Δ Δ CT^ method [24, 25] and normalized to *18s* levels [26].

### Treadmill exercise protocol

A treadmill device (MK-680, Muromachi Kikai Co, Ltd., Tokyo, Japan) was used to exercise the mice. Twenty-seven mice were randomly divided into the following nine groups (*n* = 3 mice): control (no treadmill exercise); high-intensity (18 m/min), high-frequency (every day), and long-duration (60 min/day) (HHL); high-intensity, high-frequency, and short-duration (15 min/day) (HHS); high-intensity, low-frequency (once every 3 days), and long-duration (HLL); high-intensity, low-frequency, and short-duration (HLS); low-intensity (8 m/min), high-frequency, and long-duration (LHL); low-intensity, high-frequency, and short-duration (LHS); low-intensity, low-frequency, and long-duration (LLL); and low-intensity, low-frequency, and short-duration (LLS) (Table 2). The gradient of the treadmill was graded 5% uphill. The speeds of 18 and 8 m/min correspond to running and walking for mice, respectively [27, 28]. All mice were trained on the treadmill once a day for 4 weeks after a 1-week acclimation period to treadmill exercises.

**Table 2:** Groups and exercise protocols for treadmill exercises in mice

Groups	Intensity (m/min)	Frequency	Duration (min)	Total distance (km)
Control (no treadmill exercise)	–	–	–	–
High-intensity, high-frequency, and long-duration (HHL)	18	Every day	60	30.2
High-intensity, high-frequency, and short-duration (HHS)			15	7.6
High-intensity, low-frequency, and long-duration (HLL)		Once every 3 days	60	10.1
High-intensity, low-frequency, and short-duration (HLS)			15	2.5
Low-intensity, high-frequency, and long-duration (LHL)	8	Every day	60	13.4
Low-intensity, high-frequency, and short-duration (LHS)			15	3.4
Low-intensity, low-frequency, and long-duration (LLL)		Once every 3 days	60	4.5
Low-intensity, low-frequency, and short-duration (LLS)			15	1.1

### Sampling and histological preparation

We prepared undecalcified frozen sections as previously described [29]. At the end of the experimental period, all mice were euthanized by exsanguination under anesthesia, and the knee joints were harvested. The samples were immediately freeze-embedded in 5% carboxymethyl cellulose gel. Blocks were cut into slices from the posterior of the knee joint, and 5-µm frontal sections of the proximal tibia were prepared at the level which is 40–500 μm anteriorly from a point approximately 350 μm sliced from the posterior of the knee joint (Supplementary Fig. 1). This level is the loading area of the mouse knee joint [30, 31]. All tissue staining was used on the adjacent sections spaced 20 μm apart. The right and left tibias of each animal served as different samples, and four of six tibias were randomly selected. The sample sizes were set according to Arifin et al. [32] (minimum sample size = 2.1, maximum size = 3.2) and confirmed by power analysis based on pilot results. However, a post-hoc power test on the number of chondrocytes, which is inferred to be the initial response of articular cartilage to running exercise, yielded a result of 0.29 for *n* = 3 (each of the three individuals used one leg). To ensure the reliability of the statistical results, we again confirmed the post-hoc power test with *n* = 4 (only one individual in each group randomly used both lower extremities). Accordingly, a power of 0.85 was detected; hence, the sample size was set to *n* ≥ 4. Although using both lower limbs of three individuals (*n* = 6) would have further increased detection power, we opted to use *n* = 4 given the difficultly of maintaining immunohistochemical staining consistency when more than four samples per group (36 samples in total) are used. From an ethics perspective, the use of both joints has the advantage of minimizing the number of experimental animals needed.

### Histomorphometrical analyses

We measured the articular cartilage thickness and the number of chondrocytes on digitized images of histological sections stained with safranin-O/fast green. According to our previous methods [33], the articular cartilage thickness of the tibia was measured. The cartilage thickness for each specimen was determined by averaging three sections spaced 180 μm apart.

The number of chondrocytes was quantified on sections stained with safranin-O/fast green [34]. The number of chondrocytes was manually counted using cells with visible nuclei [35].

### Histochemical and immunohistochemical analyses

Alkaline phosphatase (ALP) activity was detected according to the manufacturer’s instructions (Sigma387A-1KT, Sigma-Aldrich Japan, Tokyo, Japan), and the sections were counterstained with eosin.

According to the manufacturer’s instructions for the ApopTag^®^ Peroxidase In Situ Apoptosis Detection Kit (Chemicon S7100, Chemicon International, CA, USA), apoptosis-positive cells were detected using the TUNEL method.

According to the protocols established in our laboratory [35], sections were immunostained with antibodies against type II collagen (diluted 1:150, ab21291; Abcam, Tokyo, Japan), aggrecan (diluted 1:400, AB1031; Millipore, MA, USA), SRY-Box Transcription Factor 9 (SOX9; diluted 1:500, ab3697; Abcam), matrix metalloproteinase 13 (MMP13; diluted 1:400, ab39012; Abcam), disintegrin and metalloproteinase with thrombospondin motifs 5 (ADAMTS5; diluted 1:500, ab41037; Abcam), type X collagen (diluted 1:10,000, LB-0092; LSL, Tokyo, Japan), lubricin (diluted 1:1,500, ab28484; Abcam), and proliferating cell nuclear antigen (PCNA) (diluted 1:1000, #13,110, Cell Signaling Technology Inc., Tokyo, Japan). Subsequent reactions were performed using the streptavidin–biotin–peroxidase complex technique with an Elite ABC kit (diluted 1:50, PK-6100, Vector Laboratories, CA, USA). Immunoreactivity was visualized using 3,30-diaminobenzidine tetrahydrochloride (K3466; Dako Japan, Tokyo, Japan). We used hematoxylin (K3466, Dako Japan, Tokyo, Japan) as counterstaining to distinguish between calcified (deep zone of articular cartilage where calcified cartilage occupies more) and uncalcified regions, as previously described [36].

T. For type II collagen, histological images were converted to grayscale images using Adobe Photoshop CS2 (Adobe Systems, San Jose, CA, USA). The mean pixel gray value (in the range 0–255) was measured using Image J 1.50 (National Institutes of Health). Staining intensity was calculated using the following formula: Staining intensity = 255 – mean gray value [36].

For aggrecan, histological images were converted to grayscale images using Adobe Photoshop CS2 (Adobe Systems, San Jose, CA, USA). The immune-positive area, was measured using image J, with the maximum threshold values set at 150 for the medial non-calcified area, 170 for the lateral non-calcified area, and 140 for both calcified areas. Considering the difference in the progression of degeneration and the aggrecan content in the medial and lateral side of the knee cartilage [11, 37, 38], the maximum threshold for the medial and lateral sides was determined by examining the medial and lateral images of the tibia from one sample in each group (9 samples in total) [39]. The ratio of the immune-positive area to the articular cartilage area was then calculated.

For SOX9, lubricin, MMP13, ADAMTS5, type X collagen, ALP, PCNA, and apoptosis, the number of immune-positive cells was manually quantified.

### Statistical analyses

All statistical analyses were performed using R version 3.6.0 (R Core team). The results were compared among all groups using one-way analysis of variance (ANOVA), followed by the Tukey–Welsch test. All graphs are presented as means ± standard deviations. *P*-values < 0.05 were used to indicate statistical significance for all statistical analyses. A post hoc power analysis for the one-way ANOVA test using R was used to confirm that sufficient samples had been used (Supplementary Tables 1 and 2).

## Results

### Effects of CTS on chondrocytes

#### Anabolic gene expression

**Fig. 1:**
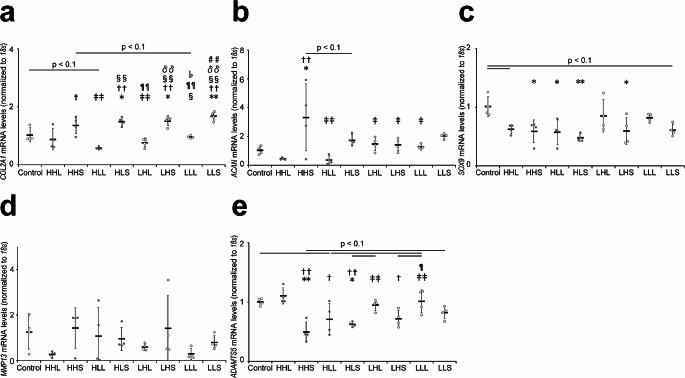
Effects of cyclic tensile strain on gene expression in mouse articular chondrocytes. The gene expression levels of (**a**) *COL2A1*, (**b**) *ACAN*, (**c**) *SOX9*, (**d**) *MMP13*, and (**e**) *ADAMTS5* were analyzed using quantitative PCR, normalized to the housekeeping gene *18s*, and relative to the control group. Untreated chondrocytes were used as the control group. Data are presented as means ± standard deviations. Statistical differences are shown as follows: *p < 0.05 vs. control; **p < 0.01 vs. control; †*p* < 0.05 vs. HHL; ††*p* < 0.01 vs. HHL; ‡*p* < 0.05 vs. HHS; ‡‡*p* < 0.01 vs. HHS; §*p* < 0.05 vs. HLL; ¶*p* < 0.05 vs. HLS; ¶¶*p* < 0.01 vs. HLS; δδ*p* < 0.01 vs. LHL; ♭*p* < 0.05 vs. LHS; ##*p* < 0.01 vs. LLL. HHL: high-intensity, high-frequency, and long-duration; HHS: high-intensity, high-frequency, and short-duration; HLL: high-intensity, low-frequency, and long-duration; HLS: high-intensity, low-frequency, and short-duration; LHL: low-intensity, high-frequency, and long-duration; LHS: low-intensity, high-frequency, and short-duration; LLL: low-intensity, low-frequency, and long-duration; LLS: low-intensity, low-frequency, and short-duration; *COL2A1*: collagen type II alpha1; *ACAN*: aggrecan, *SOX9*: sex-determining region Y-box 9; *MMT13*: matrix metallopeptidase 13; *ADAMTS5*: a disintegrin-like and metallopeptidase with thrombospondin type 1 motif 5; *18s*: 18 S ribosomal mRNA

The relative expression of *COL2A1* mRNA in the short-duration groups (i.e., HLS, LHS, and LLS groups) was significantly increased compared with that in the control group and the long-duration groups of the same intensity (Fig. 1a).

The relative expression of *ACAN* mRNA in the HHS group was significantly increased compared with that in the control and all long-duration (i.e., HHL, HLL, LHL, and LLL) groups (Fig. 1b).

The relative expression of *SOX9* mRNA in the low-intensity and long-duration (LHL and LLL) groups was significantly decreased or tended to decrease compared with that in the control group (Fig. 1c).

#### Catabolic gene expression

The relative expression of *MMP13* mRNA showed no significant difference among the groups (Fig. 1d).

The relative expression of *ADAMTS5* mRNA in the HHL group was significantly increased compared with that in all other high-intensity groups (Fig. 1e).

### Effects of treadmill exercise on the tibial cartilage

#### Cartilage thickness and number of chondrocytes

**Fig. 2:**
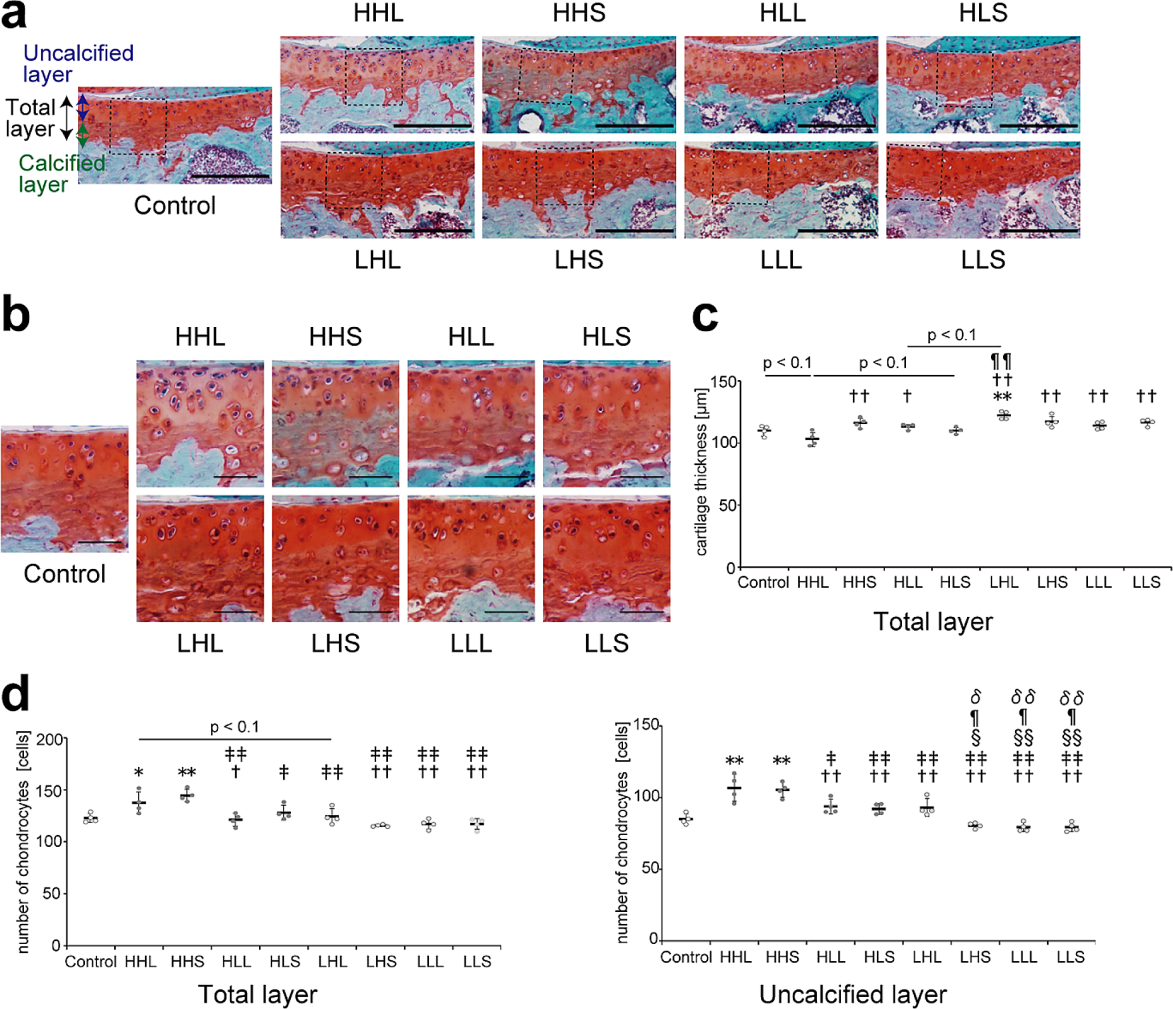
Cartilage thickness and number of chondrocytes in the tibial cartilage. (**a**) Cartilage thickness in the tibia was measured on histological sections stained with safranin O/first green. Magnified images of the boxed area in (a) are shown in (b). Scale bars = 200 μm. (**b**) Histological observations of the number of chondrocytes. Scale bars = 50 μm. (**c**) Quantitative results of the mean cartilage thickness in the total layer. (**d**) Quantitative results of the mean number of chondrocytes in the total layer. Data are presented as means ± standard deviations. Statistical differences are shown as follows: *p < 0.05 vs. control; **p < 0.01 vs. control; †*p* < 0.05 vs. HHL; ††*p* < 0.01 vs. HHL; ‡*p* < 0.05 vs. HHS; ‡‡*p* < 0.01 vs. HHS; §*p* < 0.05 vs. HLL; §§*p* < 0.01 vs. HLL; ¶*p* < 0.05 vs. HLS; ¶¶*p* < 0.01 vs. HLS; δ*p* < 0.05 vs. LHL; δδ*p* < 0.01 vs. LHL. HHL: high-intensity, high-frequency, and long-duration; HHS: high-intensity, high-frequency, and short-duration; HLL: high-intensity, low-frequency, and long-duration; HLS: high-intensity, low-frequency, and short-duration; LHL: low-intensity, high-frequency, and long-duration; LHS: low-intensity, high-frequency, and short-duration; LLL: low-intensity, low-frequency, and long-duration; LLS: low-intensity, low-frequency, and short-duration. All quantitative results (in the uncalcified, calcified, and total layers) are shown in Supplementary Fig. 2

The cartilage surface was intact without cartilage damage, and no obvious difference in cartilage matrix staining to safranin as a reflection of proteoglycan content was observed between all groups (Fig. 2a). The cartilage thickness in the total layer in the HHL group was significantly decreased or tended to decrease compared with that in all groups (Fig. 2c). Furthermore, the number of chondrocytes in the total layer in the HHL group was significantly increased compared with that in the control group, particularly in the uncalcified layer in the HHL group, which was significantly increased compared with that in all groups, except for the HHS group (Fig. 2b and d). In contrast, the cartilage thickness in the total layer in the LHL group was significantly increased compared with that in the control, HHL, and HLS groups or tended to increase compared with that in the HLL group, although no significant differences were observed among the low-intensity groups (Fig. 2c). The number of chondrocytes in the uncalcified layer in the LHL group was significantly increased compared with that in all other low-intensity groups (Fig. 2b and d).

Consistent with the increase in the number of chondrocytes in the HHL group, the percentage of PCNA-positive cells, as a marker of cell proliferation, in the total and uncalcified layers in the HHL group was significantly increased compared with that in all groups, except for the HHS group (Supplementary Fig. 3).

An increase in cartilage thickness with increased matrix synthesis is beneficial to articular cartilage, whereas an increase in cartilage thickness with cellular activities, such as chondrocyte hypertrophy, may be harmful to articular cartilage [40]. Therefore, we next evaluated protein changes associated with the cartilage matrix and chondrocyte hypertrophy.

### Markers of inhibitory effect on chondrocyte hypertrophy

**Fig. 3:**
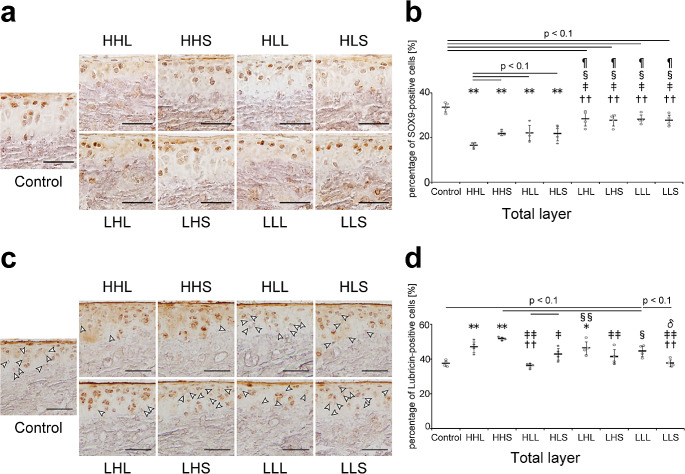
Representative images of the tibial cartilage stained with (a) SOX9 and (c) lubricin and the quantitative results. (**a**) Scale bars = 40 μm. (**b**) Quantitative results of the percentage of SOX9-positive cells in the total layer. Data are presented as means ± standard deviations. (**c**) White arrowheads represented Lubricin-negative cells. Scale bars = 50 μm. (**d**) Quantitative results of the percentage of lubricin-negative cells in the total layer. Data are presented as means ± standard deviations. Statistical differences are shown as follows: *p < 0.05 vs. control; **p < 0.01 vs. control; ††*p* < 0.01 vs. HHL; ‡*p* < 0.05 vs. HHS; ‡‡*p* < 0.01 vs. HHS; §*p* < 0.05 vs. HLL; §§*p* < 0.01 vs. HLL; ¶*p* < 0.05 vs. HLS; δ*p* < 0.05 vs. LHL. HHL: high-intensity, high-frequency, and long-duration; HHS: high-intensity, high-frequency, and short-duration; HLL: high-intensity, low-frequency, and long-duration; HLS: high-intensity, low-frequency, and short-duration; LHL: low-intensity, high-frequency, and long-duration; LHS: low-intensity, high-frequency, and short-duration; LLL: low-intensity, low-frequency, and long-duration; LLS: low-intensity, low-frequency, and short-duration; SOX9: sex-determining region Y-box 9. All quantitative results of SOX9 (in the uncalcified, calcified, and total layers) are shown in Supplementary Fig. 4a

SOX9-positive cells, which contribute to the suppression of chondrocyte hypertrophy and the maintenance of cartilage homeostasis [41], were observed in both the uncalcified and calcified layers (Fig. 3a). The percentage of SOX9-positive cells in the total layer in all high-intensity groups was significantly decreased compared with that in the control and low-intensity groups, and this decrease was more pronounced in the HHL group (Fig. 3a and b).

Lubricin, which maintains cartilage integrity [42] and inhibits hypertrophy and catabolism in the cartilage [43], was localized on the cartilage surface and within chondrocytes in the uncalcified layer (Fig. 3c). The percentage of lubricin-positive cells in the high-intensity and high-frequency (HHL and HHS) and low-intensity and long-duration (LHL and LLL) groups was significantly increased or tended to increase compared with that in the control group (Fig. 3c and d). For the low-intensity groups, the percentage of lubricin-positive cells in the LHL and LLL groups was significantly increased or tended to increase compared with that in the LLS group (Fig. 3c and d).

### Cartilage matrix and proteases

**Fig. 4:**
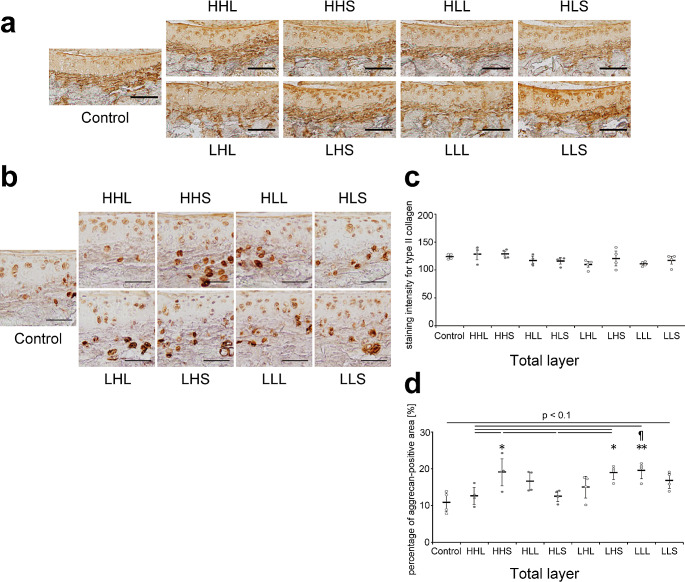
Representative images of the tibial cartilage stained with (**a**) type II collagen and (b) aggrecan and the quantitative results. (a) Scale bars = 100 μm. (**b**) Scale bars = 50 μm. (**c**) Quantitative results of staining intensity for type II collagen in the total layer. Data are presented as means ± standard deviations. (**d**) Quantitative results of the percentage of aggrecan-positive area in the total layer. Data are presented as means ± standard deviations. Statistical differences are shown as follows: *p < 0.05 vs. control; **p < 0.01 vs. control; ¶*p* < 0.05 vs. HLS. HHL: high-intensity, high-frequency, and long-duration; HHS: high-intensity, high-frequency, and short-duration; HLL: high-intensity, low-frequency, and long-duration; HLS: high-intensity, low-frequency, and short-duration; LHL: low-intensity, high-frequency, and long-duration; LHS: low-intensity, high-frequency, and short-duration; LLL: low-intensity, low-frequency, and long-duration; LLS: low-intensity, low-frequency, and short-duration. All quantitative results (in the uncalcified, calcified, and total layers) are shown in Supplementary Fig. 4b and 4c

Type II collagen was evenly distributed throughout the cartilage, and staining intensity showed no significant differences among all groups (Fig. 4a and c). Aggrecan, which is the major proteoglycan in articular cartilage, was uniform across the entire cartilage and localized within or around chondrocytes (Fig. 4b). The percentage of aggrecan-positive areas in the total layer in the HHS, LHS, and LLL groups was significantly increased compared with that in the control group and tended to increase compared with that in the HHL group (Fig. 4b and d).

**Fig. 5:**
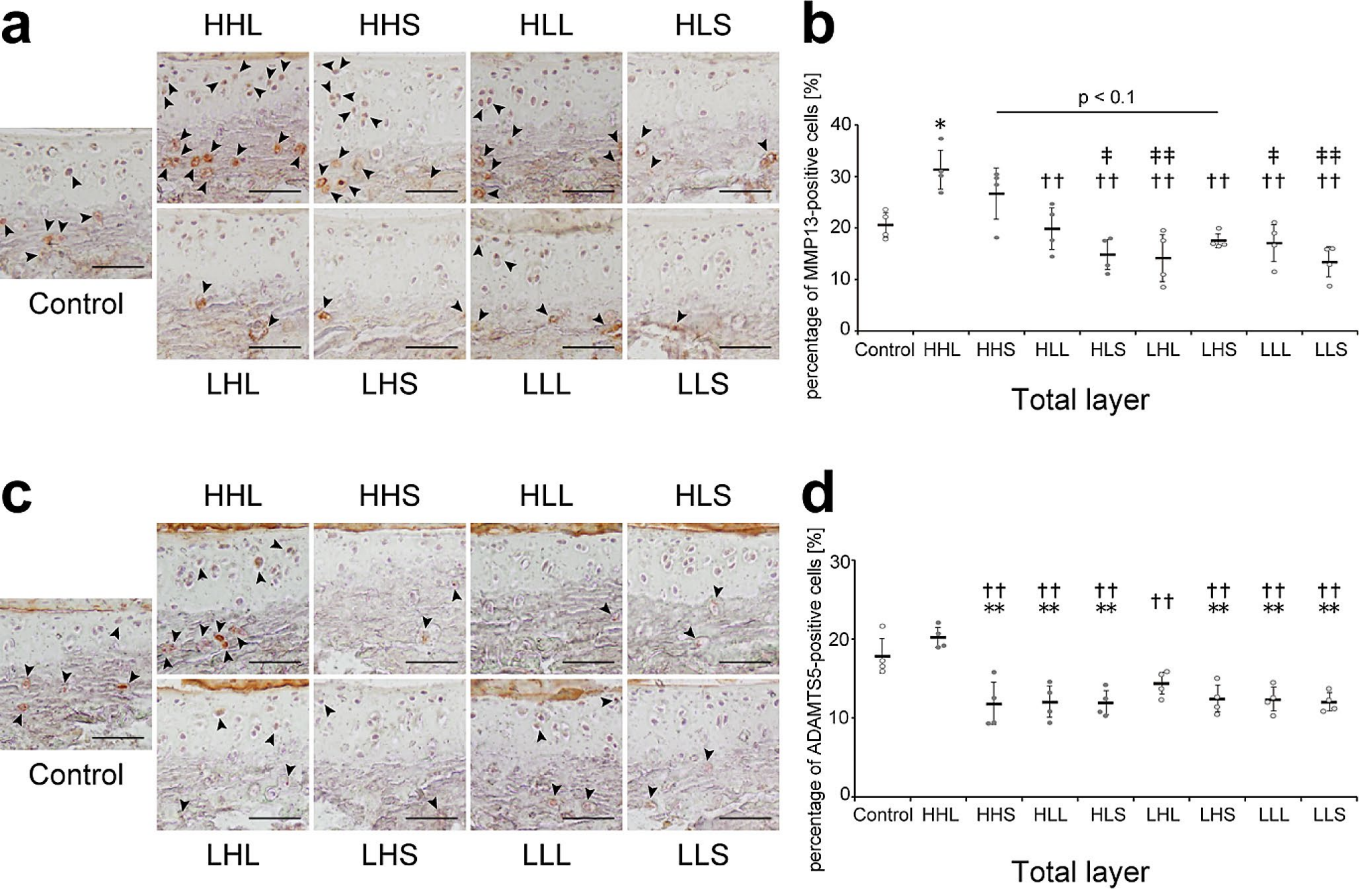
Representative images of the tibial cartilage stained with (a) MMP13 and (c) ADAMTS5 and the quantitative results. (**a**) Black arrowheads represented MMP13-positive cells. Scale bars = 50 μm. (**b**) Quantitative results of the percentage of MMP13-positive cells in the total layer. Data are presented as means ± standard deviations. (**c**) Black arrowheads represented ADAMTS5-positive cells. Scale bars = 50 μm. (**d**) Quantitative results of the percentage of ADAMTS5-positive cells in the total layer. Data are presented as means ± standard deviations. Statistical differences are shown as follows: *p < 0.05 vs. control; **p < 0.01 vs. control; ††*p* < 0.01 vs. HHL; ‡*p* < 0.05 vs. HHS; ‡‡*p* < 0.01 vs. HHS. HHL: high-intensity, high-frequency, and long-duration; HHS: high-intensity, high-frequency, and short-duration; HLL: high-intensity, low-frequency, and long-duration; HLS: high-intensity, low-frequency, and short-duration; LHL: low-intensity, high-frequency, and long-duration; LHS: low-intensity, high-frequency, and short-duration; LLL: low-intensity, low-frequency, and long-duration; LLS: low-intensity, low-frequency, and short-duration; MMT13: matrix metallopeptidase 13; ADAMTS5: a disintegrin-like metallopeptidase with thrombospondin type 1 motif 5. All quantitative results (in the uncalcified, calcified, and total layers) are shown in Supplementary Fig. 5a and 5b

MMP13-positive cells, the most active in cleaving type II collagen, were mainly observed within chondrocytes in the calcified layer, whereas, in the high-intensity and high-frequency (HHL and HHS) groups, these cells were also detected in chondrocytes in the uncalcified layer (Fig. 5a). The percentage of MMP13-positive cells in the total layer in the HHL group was significantly increased compared with that in all groups, except for the HHS group (Fig. 5a and b). ADAMTS5-positive cells, the major aggrecanase in mouse cartilage, were mainly observed within chondrocytes in the calcified layer (Fig. 5c). The percentage of ADAMTS5-positive cells in the total layer in almost all exercise groups (except for LHL group) was significantly decreased compared with that in the control and HHL groups (Fig. 5c and d).

### Markers of chondrocyte hypertrophy

**Fig. 6:**
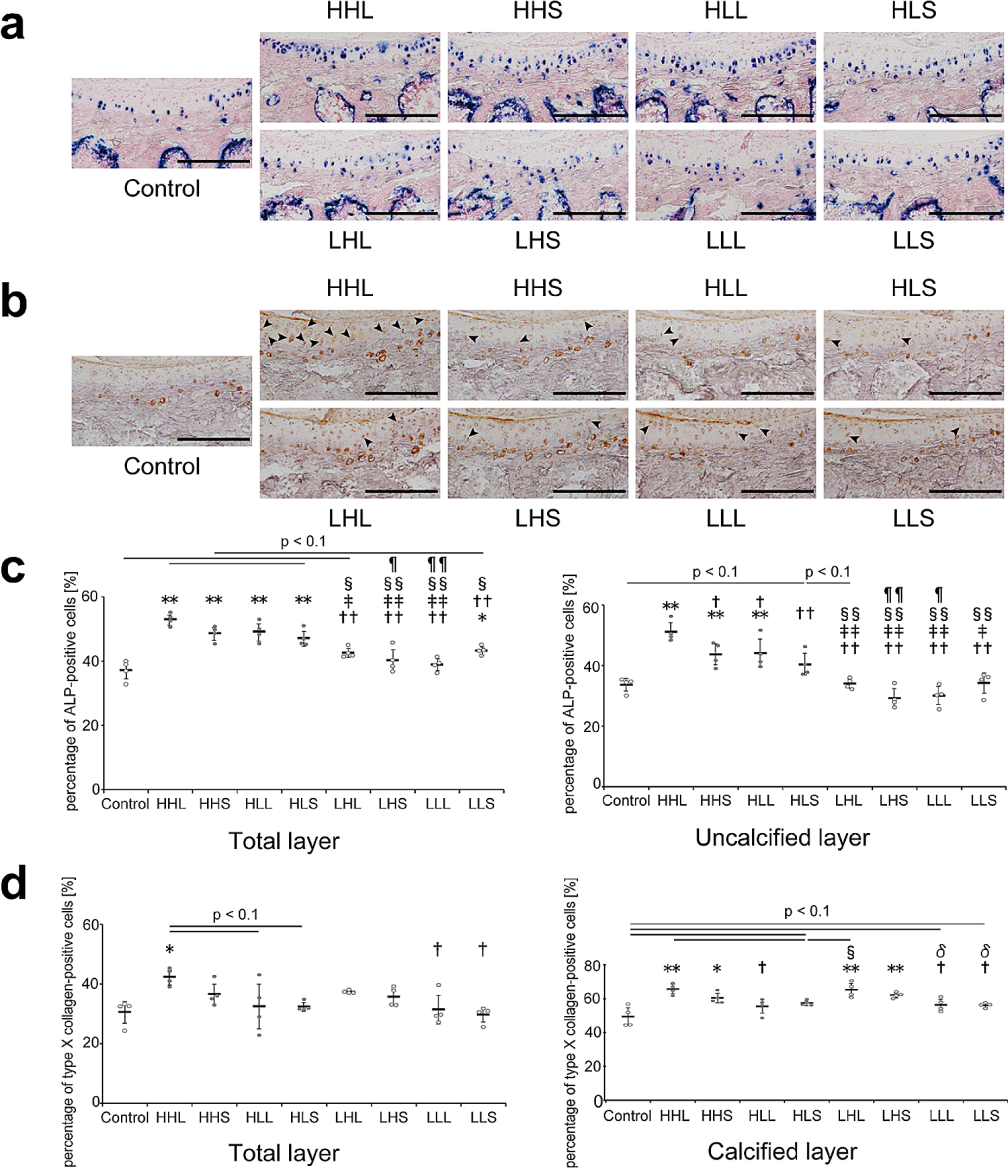
Representative images of the tibial cartilage stained with (a) ALP and (b) type X collagen and the quantitative results. (**a**) Scale bars = 200 μm. (**b**) Type X collagen-positive cells in the uncalcified layer are represented by arrowheads. Scale bars = 200 μm. (**c**) Quantitative results of the percentage of ALP-positive cells in the total and uncalcified layers. Data are presented as means ± standard deviations. (**d**) Quantitative results of the percentage of type X collagen-positive cells in the total and calcified layers. Data are presented as means ± standard deviations. Statistical differences are shown as follows: *p < 0.05 vs. control; **p < 0.01 vs. control; †*p* < 0.05 vs. HHL; ††*p* < 0.01 vs. HHL; ‡*p* < 0.05 vs. HHS; ‡‡*p* < 0.01 vs. HHS; §*p* < 0.05 vs. HLL; §§*p* < 0.01 vs. HLL; ¶*p* < 0.05 vs. HLS; ¶¶*p* < 0.01 vs. HLS; δ*p* < 0.05 vs. LHL. HHL: high-intensity, high-frequency, and long-duration; HHS: high-intensity, high-frequency, and short-duration; HLL: high-intensity, low-frequency, and long-duration; HLS: high-intensity, low-frequency, and short-duration; LHL: low-intensity, high-frequency, and long-duration; LHS: low-intensity, high-frequency, and short-duration; LLL: low-intensity, low-frequency, and long-duration; LLS: low-intensity, low-frequency, and short-duration; ALP: alkaline phosphatase. All quantitative ALP results (in the uncalcified, calcified, and total layers) are shown in Supplementary Fig. 5c

ALP-positive cells, which are a marker of maturing chondrocytes [44], were located on the tidemark and in the calcified cartilage in all groups (Fig. 6a). The percentage of ALP-positive cells in the uncalcified layer in all high-intensity groups was significantly increased or tended to increase compared with that in the control group (Fig. 6c). Furthermore, the percentage of ALP-positive cells in the uncalcified layer in all high-intensity groups (except for the HLS group vs. LLS group) was significantly increased or tended to increase compared with that in the low-intensity groups (Fig. 6c).

Type X collagen-positive cells, which are a specific marker of chondrocyte hypertrophy, were observed in the calcified layer in all groups, and an increase in type X collagen-positive cells in the uncalcified layer was observed in the HHL group (Fig. 6b). The percentage of type X collagen-positive cells in the total layer was significantly increased only in the HHL group compared with that in the control group (Fig. 6d). The percentage of type X collagen-positive cells in the calcified layer in the high-frequency (i.e., HHL, HHS, LHL, and LHS) groups was significantly increased, regardless of the intensity, compared with that in the control group (Fig. 6d). Furthermore, for the low-intensity groups, the percentage of type X collagen-positive cells in the calcified layer in the LHL group was significantly higher than that in the low-frequency (LLL and LLS) groups (Fig. 6d).

Furthermore, the percentage of TUNEL-positive cells, which indicates chondrocyte apoptosis [45], in the total layer in the high-intensity and high-frequency (HHL and HHS) groups was significantly increased compared with that in the control and low-intensity groups (Supplementary Fig. 6a and 6b).

## Discussion

Our objective was to determine the combination of intensity, frequency, and duration that provides the best mechanical loading on healthy knee articular cartilage. We found that low-intensity CTS, regardless of frequency, activated anabolic gene expression in the cartilage matrix during the short duration and suppressed the downregulation of gene expression of *SOX9* during the long duration. Furthermore, low-intensity, low-frequency, and long-duration treadmill exercises inhibited chondrocyte hypertrophy and increased aggrecan synthesis in the tibial cartilage. Madden et al. [46] reported that 10–20% compressive stress on articular cartilage, which can be considered a physiological load, causes chondrocyte elongation of approximately 5–13%. Moreover, another review found that in vivo chondrocytes are not subjected to > 15% cell elongation [8]. In previous studies, slow walking was assumed to create loads between 0.5 and < 1.0 Hz, while jogging or running creates loads above 1.0 Hz [47–49]. Given that the current study used both in vivo and 2D in vitro culture experiments, the loading on the articular cartilage of the knee joint during in vivo walking and running experiments cannot be directly linked to chondrocyte-loading parameters during in vitro experiments. However, given that walking and running encompass the range of physiological motion, in vitro chondrocyte-loading parameters were also designed to be within the range expected during in vivo walking and running.

Chondrocytes adapt to changes in the mechanical environment by upregulating anabolic genes. The expression of these genes is then downregulated [8, 50]. Reports have shown that chondrocyte mRNA response to CTS is immediate in some cases but delayed in others, and that responses decrease with time [51]. Similarly, other studies, such as those by our group, also investigated the response of mRNA expression over time [52]. Notably, available evidence suggests that the 12-h timepoint can be considered the approximate time point at which the response of chondrocytes to CTS switches from anabolism to catabolism [8]. However, *ACAN* mRNA did not show time-dependent changes similar to *COL2A1* mRNA. This poor response may be the reason that proteoglycan loss precedes type II collagen loss in early cartilage degeneration [53]. *SOX9* mRNA, which contributes to cartilage homeostasis [41], was downregulated in the high-intensity groups, similar to the findings of previous studies [54, 55]. However, interestingly, this downregulation also occurred in the low-intensity and short-duration groups, and the expression of *SOX9* mRNA in the low-intensity and long-duration groups was maintained at the same level compared with that in the control group. *TGF-β*, which inhibits chondrocyte hypertrophy, contributes to the expression of *SOX9* [56], and *TGF-β* mRNA is upregulated when CTS is applied with intensity of 5–12% and duration of 12–24 h [57, 58]. These differences suggest that mRNA responses may be altered by differences in combination with stimulus intensity and frequency, not only duration. Taken together, low-intensity CTS applied for > 12 h may be helpful to maintain cartilage homeostasis via *SOX9*.

Histological analysis after treadmill exercises revealed that mechanical loading in the HHL group had the most negative effects on healthy articular cartilage, and these findings are consistent with those of a previous review [9]. The increase in cartilage thickness with the increase in chondrocyte proliferation and hypertrophy, which were observed in the LHL group, have been reported as precursors to cartilage degeneration [40, 59, 60]. In contrast, although the cartilage thickness was similar to that in the control group, only the cartilage in the LLL group exhibited an increase in aggrecan and lubricin without an increase in chondrocyte hypertrophy compared with that in the control group, and these responses may maintain/improve the loading function of articular cartilage.

Because high-frequency exercises resulted in catabolism, including chondrocyte hypertrophy, even in the low-intensity groups, daily exercises without rest may lead to chondrocyte hypertrophy [61]. In contrast, cartilage in the LLS group did not change. This is consistent with the findings of previous studies, which have reported that mechanical loading loads over a certain amount of time and frequency are necessary to maintain articular cartilage health [8, 16].

This study has several limitations. First, the best mechanical loading on the articular cartilage presented in this study was achieved by combining large or small values for each parameter, but not specific values. Although further research is required, our findings provide useful insights for future research. Directly linking CTS to treadmill exercise results may be difficult because the load on the knee joint caused by treadmill exercises does not reflect the exact amount of mechanical loading on the cartilage or chondrocytes [61]. Interestingly, low-intensity and long-duration mechanical loading was a suitable combination for the articular cartilage in both the CTS and treadmill exercise results.

## Conclusions

Our results show that low-intensity, low-frequency, and long-duration mechanical loading is the best combination for healthy knee articular cartilage to maintain homeostasis. Our results may provide a significant scientific basis for designing exercise programs and lifestyle instructions that consider the mechanical loading on articular cartilage.

## Electronic supplementary material

Below is the link to the electronic supplementary material.

Supplementary Material 1

## Data Availability

No datasets were generated or analysed during the current study.
