# Supplementary material for: Effects of different combinations of mechanical loading intensity, duration, and frequency on the articular cartilage in mice
Source: Mol Biol Rep. 2024 Jul 29;51(1):862. doi: 10.1007/s11033-024-09762-5 (PMC11286701; doi:10.1007/s11033-024-09762-5)
Supplement: Supplementary file 1 — Supplementary Material 1 [file 11033_2024_9762_MOESM1_ESM.docx]

**Supplementary Information**

**Supplementary Table 1** Statistical results of the one-way analysis of variance test *in vitro* (real-time PCR)

|  | ***P*-value** | **Effect size** | **Statistical power** |
| --- | --- | --- | --- |
| COL2A1 | <0.001 | 1.85 | 1 |
| ACAN | 0.001 | 1.07 | 0.99 |
| SOX9 | 0.003 | 0.99 | 0.98 |
| MMP13 | 0.287 | 0.57 | 0.53 |
| ADAMTS5 | <0.001 | 1.37 | 1 |

**Supplementary Table 2** Statistical results of the one-way analysis of variance test *in vivo* (histological and immunohistochemical analyses)

|  |  | ***P*-value** | **Effect size** | **Statistical power** |
| --- | --- | --- | --- | --- |
| Cartilage thickness | Uncalcified layer | 0.612 | 0.45 | 0.33 |
|  | Calcified layer | <0.001 | 1.40 | 1 |
|  | Total layer | <0.001 | 1.43 | 1 |
| Number of chondrocytes | Uncalcified layer | <0.001 | 2.06 | 1 |
|  | Calcified layer | 0.038 | 0.79 | 0.85 |
|  | Total layer | <0.001 | 1.55 | 1 |
| PCNA | Uncalcified layer | <0.001 | 1.32 | 1 |
|  | Calcified layer | 0.507 | 0.48 | 0.38 |
|  | Total layer | <0.001 | 1.11 | 0.99 |
| SOX9 | Uncalcified layer | <0.001 | 1.24 | 1 |
|  | Calcified layer | <0.001 | 2.30 | 1 |
|  | Total layer | <0.001 | 1.81 | 1 |
| Lubricin | – | <0.001 | 1.60 | 1 |
| Type II collagen | Uncalcified layer | 0.281 | 0.57 | 0.54 |
|  | Calcified layer | 0.025 | 0.82 | 0.89 |
|  | Total layer | 0.120 | 0.67 | 0.70 |
| Aggrecan | Uncalcified layer | 0.057 | 0.75 | 0.81 |
|  | Calcified layer | <0.001 | 1.41 | 1 |
|  | Total layer | <0.001 | 1.12 | 1 |
| MMP13 | Uncalcified layer | <0.001 | 1.89 | 1 |
|  | Calcified layer | 0.020 | 0.84 | 0.91 |
|  | Total layer | <0.001 | 1.47 | 1 |
| ADAMTS5 | Uncalcified layer | 0.005 | 0.96 | 0.97 |
|  | Calcified layer | <0.001 | 2.34 | 1 |
|  | Total layer | <0.001 | 1.50 | 1 |
| ALP | Uncalcified layer | <0.001 | 2.01 | 1 |
|  | Calcified layer | <0.001 | 1.36 | 1 |
|  | Total layer | <0.001 | 2.04 | 1 |
| Type X collagen | Uncalcified layer | 0.014 | 0.87 | 0.93 |
|  | Calcified layer | <0.001 | 1.41 | 1 |
|  | Total layer | 0.007 | 0.93 | 0.96 |
| TUNEL | Uncalcified layer | <0.001 | 1.45 | 1 |
|  | Calcified layer | 0.012 | 0.89 | 0.94 |
|  | Total layer | <0.001 | 1.48 | 1 |

**Supplementary Figure**


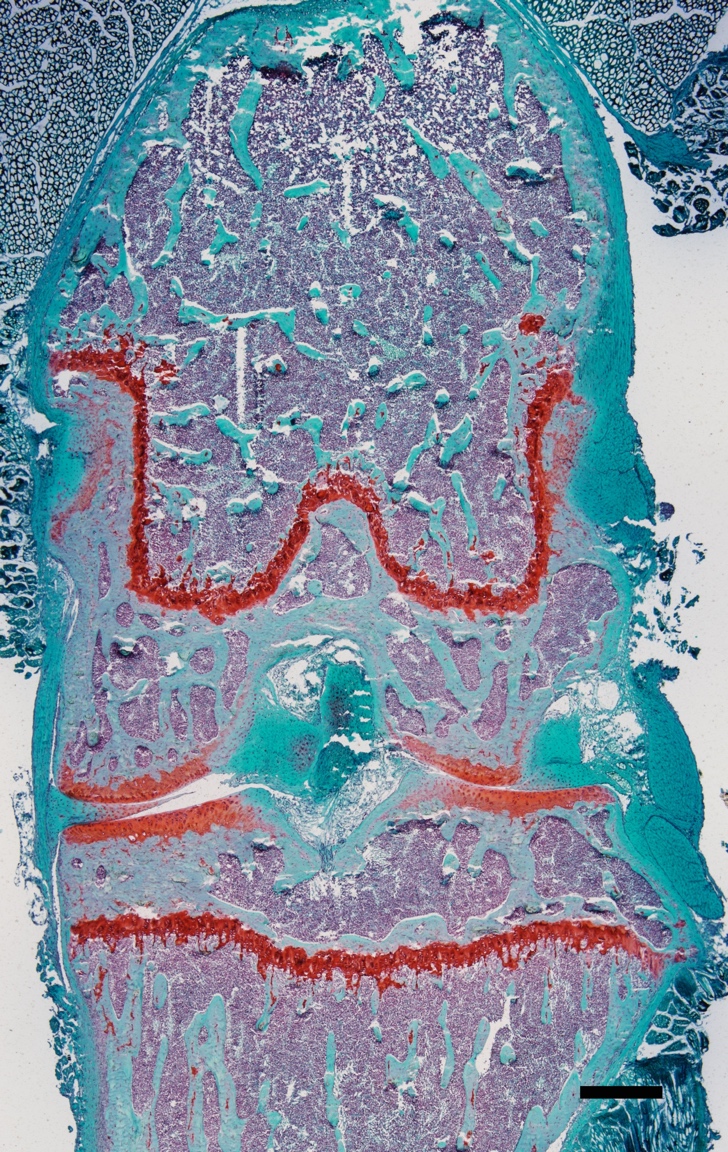


**Supplementary Fig. 1** The whole image of the mouse knee joint frontal section from the control group. This level is 260 μm anteriorly from a point approximately 350 μm sliced from the posterior of the knee joint. Scale bars = 400 μm


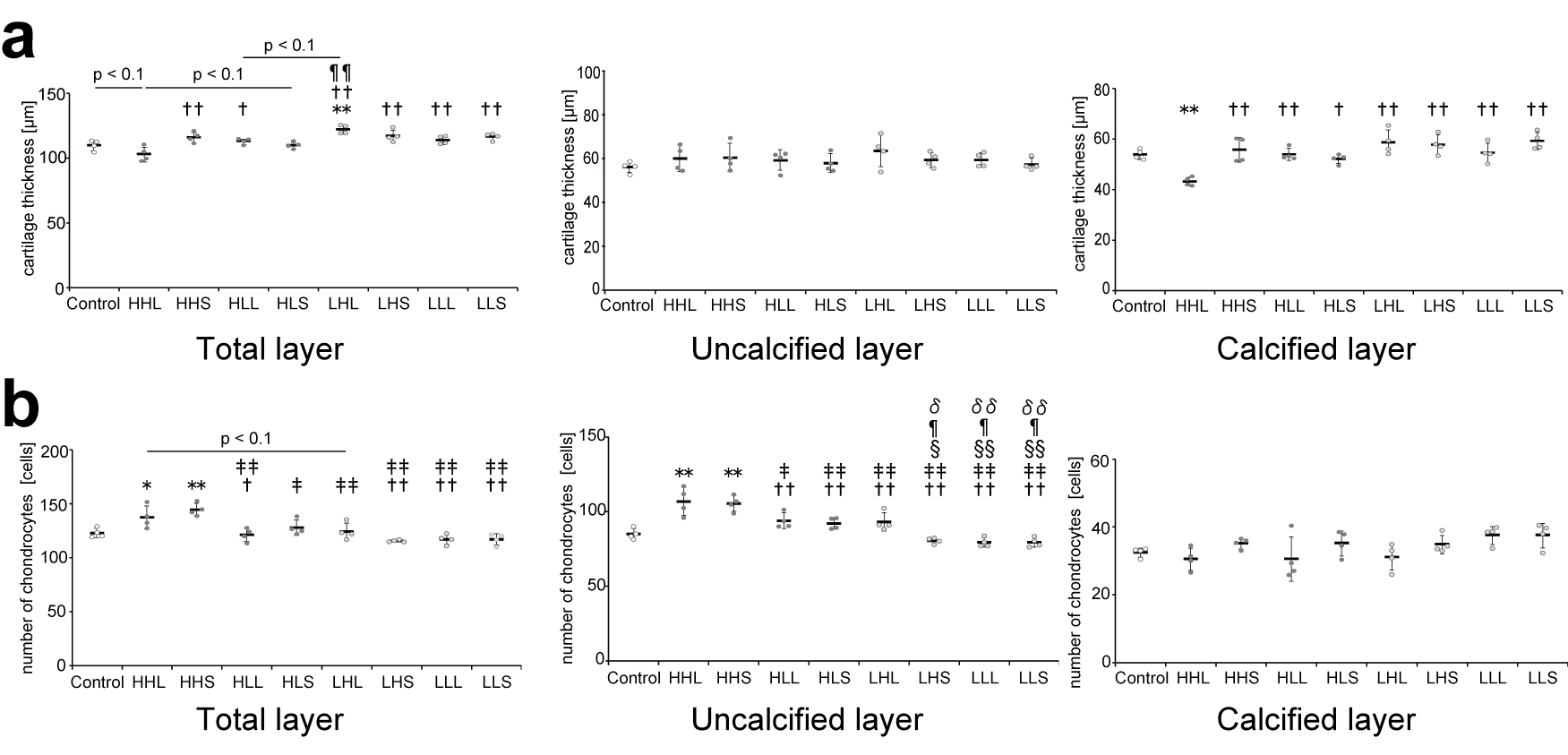


**Supplementary Fig. 2** Cartilage thickness and number of chondrocytes in the tibial cartilage. (a) Thicknesses in the uncalcified, calcified, and total layers of the tibial cartilage were measured separately on histological sections stained with safranin O/first green. Quantitative results of the mean cartilage thickness of the three regions. (b) Quantitative results of the mean number of chondrocytes of the three regions. Data are presented as means ± standard deviations. Statistical differences are shown as follows: **p* < 0.05 *vs.* control; ***p* < 0.01 *vs.* control; †*p* < 0.05 *vs.* HHL; ††*p* < 0.01 *vs.* HHL; ‡*p* < 0.05 *vs.* HHS; ‡‡*p* < 0.01 *vs.* HHS; §*p* < 0.05 *vs.* HLL; §§*p* < 0.01 *vs.* HLL; ¶*p* < 0.05 *vs.* HLS; ¶¶*p* < 0.01 *vs.* HLS; δ*p* < 0.05 *vs.* LHL; δδ*p* < 0.01 *vs.* LHL. HHL: high-intensity, high-frequency, and long-duration; HHS: high-intensity, high-frequency, and short-duration; HLL: high-intensity, low-frequency, and long-duration; HLS: high-intensity, low-frequency, and short-duration; LHL: low-intensity, high-frequency, and long-duration; LHS: low-intensity, high-frequency, and short-duration; LLL: low-intensity, low-frequency, and long-duration; LLS: low-intensity, low-frequency, and short-duration


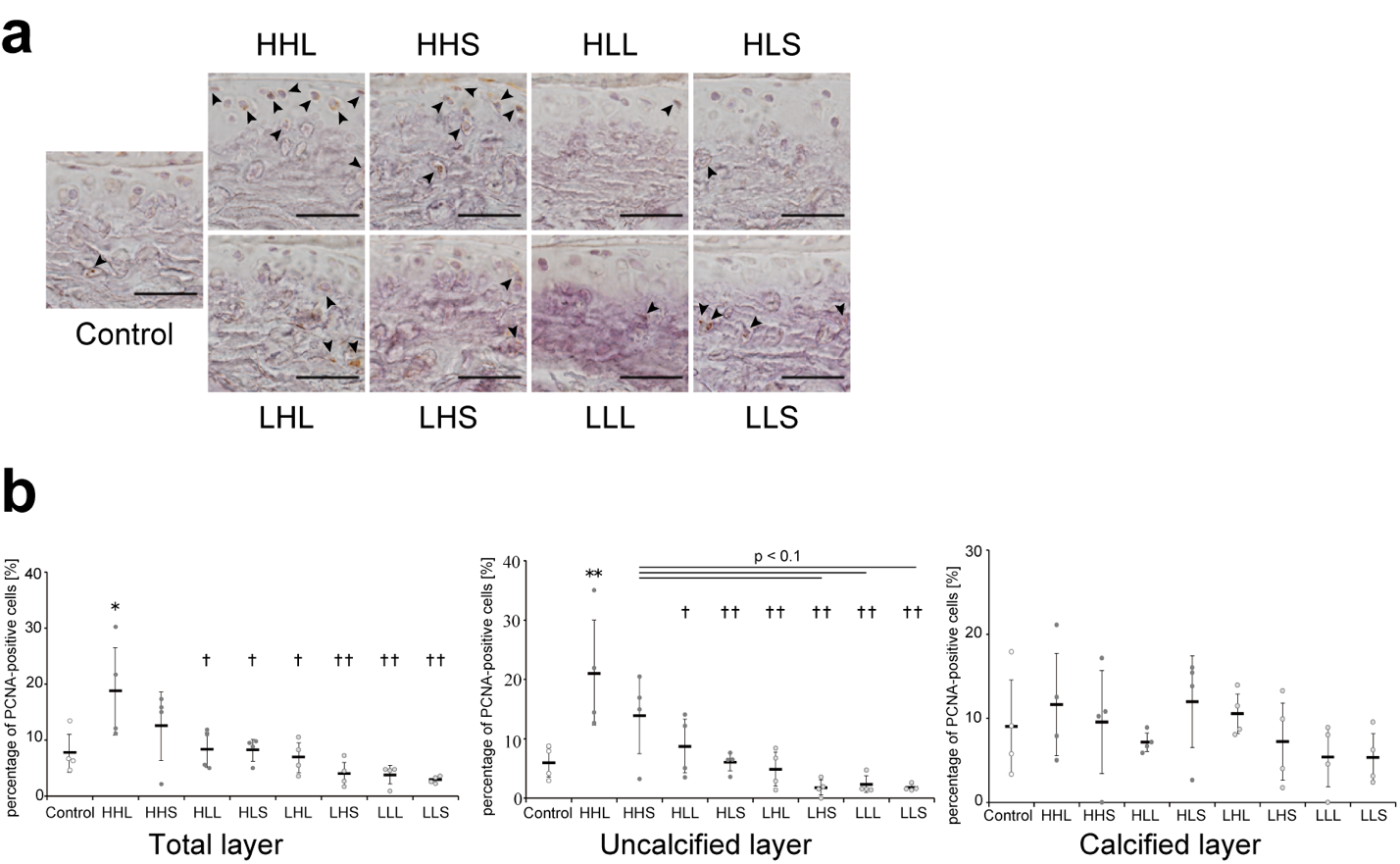


**Supplementary Fig. 3** Representative image of the tibial cartilage stained with PCNA and the quantitative results. (a) Black arrowheads represent positive cells. Scale bars = 40 μm. (b) Quantitative results of the percentage of PCNA-positive cells. Data are presented as means ± standard deviations. Statistical differences are shown as follows: **p* < 0.05 *vs.* control; ***p* < 0.01 *vs.* control; †*p* < 0.05 *vs.* HHL; ††*p* < 0.01 *vs.* HHL. HHL: high-intensity, high-frequency, and long-duration; HHS: high-intensity, high-frequency, and short-duration; HLL: high-intensity, low-frequency, and long-duration; HLS: high-intensity, low-frequency, and short-duration; LHL: low-intensity, high-frequency, and long-duration; LHS: low-intensity, high-frequency, and short-duration; LLL: low-intensity, low-frequency, and long-duration; LLS: low-intensity, low-frequency, and short-duration; PCNA: proliferating cell nuclear antigen


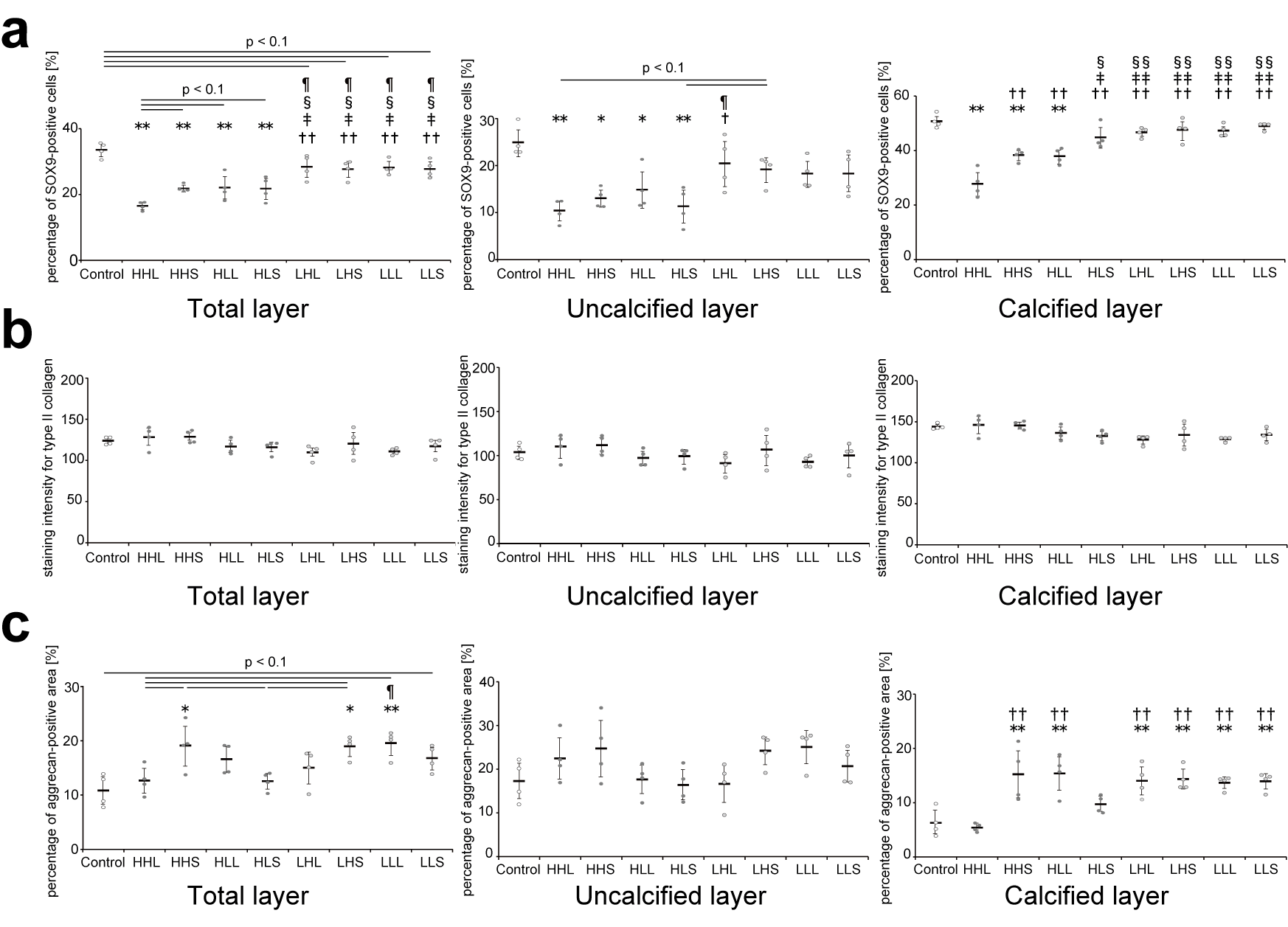


**Supplementary Fig. 4** Quantitative results of (a) the percentage of SOX9-positive cells, (b) staining intensity for type II collagen, and (c) the percentage of aggrecan-positive area in the articular cartilage. Data are presented as means ± standard deviations. Statistical differences are shown as follows: **p* < 0.05 *vs.* control; ***p* < 0.01 *vs.* control; †*p* < 0.05 *vs.* HHL; ††*p* < 0.01 *vs.* HHL; ‡*p* < 0.05 *vs.* HHS; ‡‡*p* < 0.01 *vs.* HHS; §*p* < 0.05 *vs.* HLL; §§*p* < 0.01 *vs.* HLL; ¶*p* < 0.05 *vs.* HLS. HHL: high-intensity, high-frequency, and long-duration; HHS: high-intensity, high-frequency, and short-duration; HLL: high-intensity, low-frequency, and long-duration; HLS: high-intensity, low-frequency, and short-duration; LHL: low-intensity, high-frequency, and long-duration; LHS: low-intensity, high-frequency, and short-duration; LLL: low-intensity, low-frequency, and long-duration; LLS: low-intensity, low-frequency, and short-duration. SOX9: sex-determining region Y-box 9


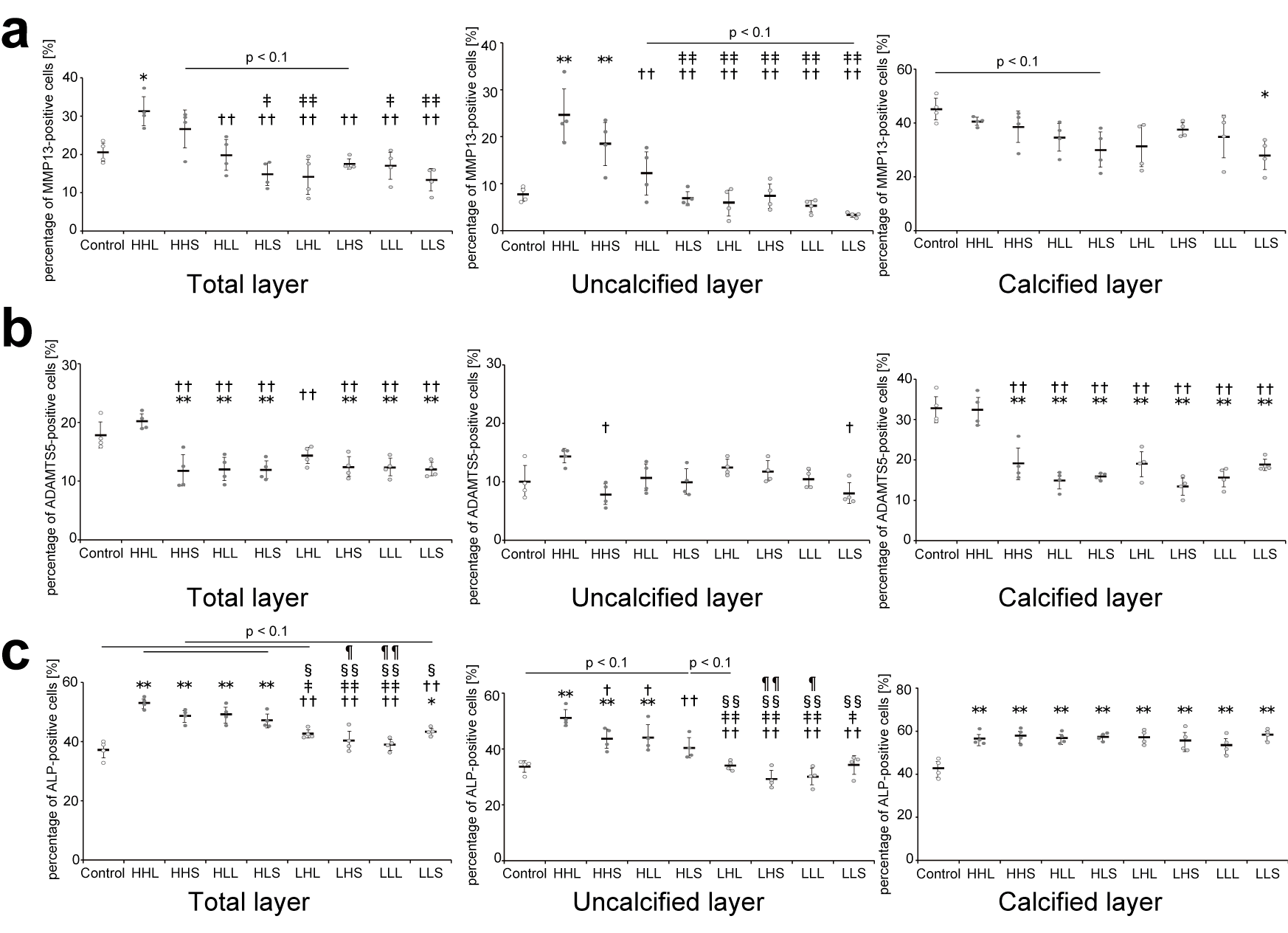


**Supplementary Fig. 5** Quantitative results of (a) the percentage of MMP13-positive cells, (b) percentage of ADAMTS5-positive cells, and (c) percentage of ALP-positive cells in the articular cartilage. Data are presented as means ± standard deviations. Statistical differences are shown as follows: **p* < 0.05 *vs.* control; ***p* < 0.01 *vs.* control; †*p* < 0.05 *vs.* HHL; ††*p* < 0.01 *vs.* HHL; ‡*p* < 0.05 *vs.* HHS; ‡‡*p* < 0.01 *vs.* HHS; §*p* < 0.05 *vs.* HLL; §§*p* < 0.01 *vs.* HLL; ¶*p* < 0.05 *vs.* HLS; ¶¶*p* < 0.01 *vs.* HLS. HHL: high-intensity, high-frequency, and long-duration; HHS: high-intensity, high-frequency, and short-duration; HLL: high-intensity, low-frequency, and long-duration; HLS: high-intensity, low-frequency, and short-duration; LHL: low-intensity, high-frequency, and long-duration; LHS: low-intensity, high-frequency, and short-duration; LLL: low-intensity, low-frequency, and long-duration; LLS: low-intensity, low-frequency, and short-duration; MMT13: matrix metallopeptidase 13; ADAMTS5: a disintegrin-like and metallopeptidase with thrombospondin type 1 motif 5; ALP: alkaline phosphatase


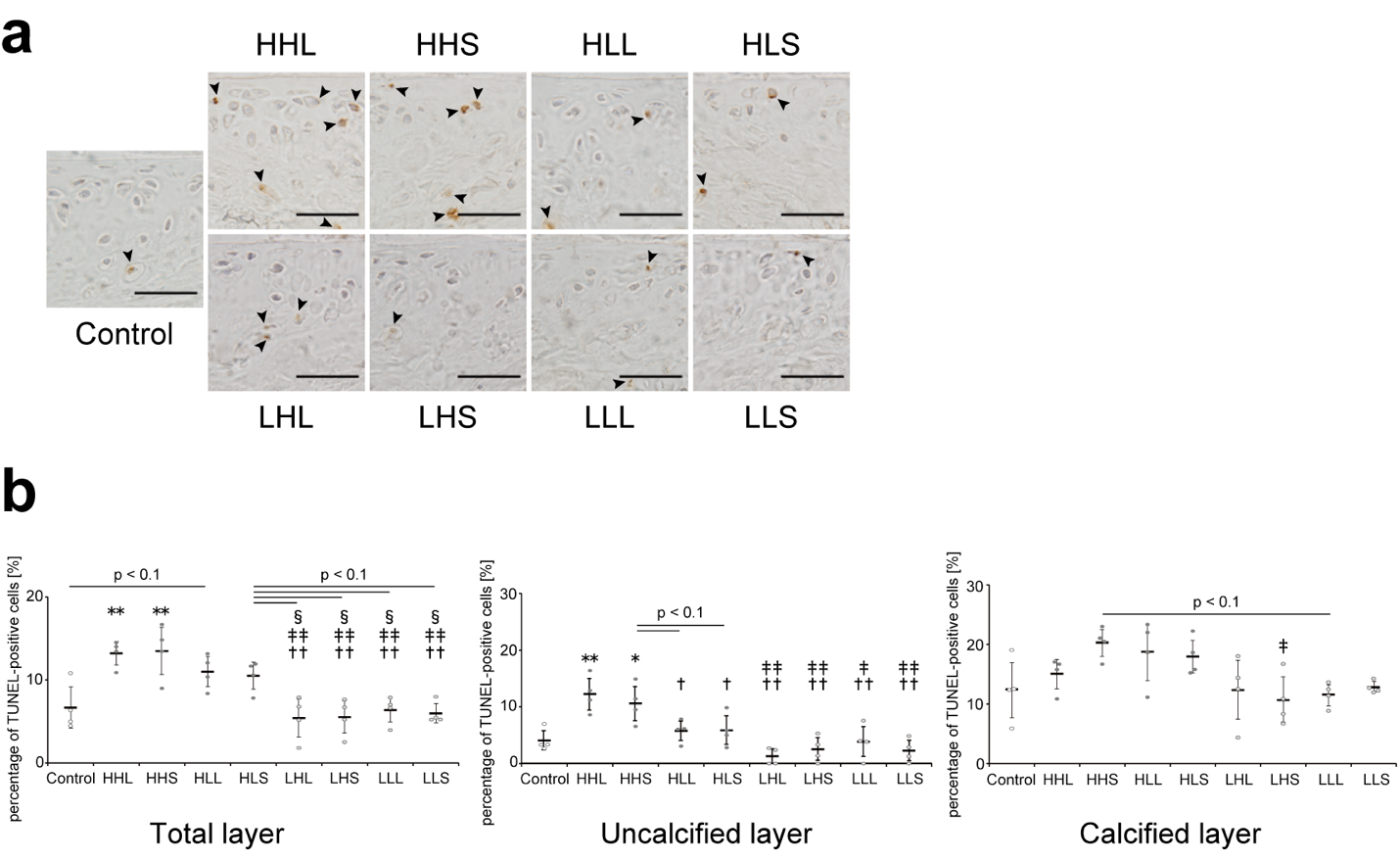


**Supplementary Fig. 6** Representative image of the tibial cartilage stained with TUNEL assay and the quantitative results. (a) TUNEL-positive cells were slightly observed in the mainly calcified layer in the control and low-intensity groups, whereas, in the high-intensity (particularly, HHL and HHS) groups, TUNEL-positive cells were also observed in the noncalcified layer. Black arrowheads represent TUNEL-positive cells. Scale bars = 40 μm. (b) Quantitative results of the percentage of TUNEL-positive cells. Data are presented as means ± standard deviations. Statistical differences are shown as follows: **p* < 0.05 *vs.* control; ***p* < 0.01 *vs.* control; ††*p* < 0.01 *vs.* HHL; ‡*p* < 0.05 *vs.* HHS; ‡‡*p* < 0.01 *vs.* HHS; §*p* < 0.05 *vs.* HLL. HHL: high-intensity, high-frequency, and long-duration; HHS: high-intensity, high-frequency, and short-duration; HLL: high-intensity, low-frequency, and long-duration; HLS: high-intensity, low-frequency, and short-duration; LHL: low-intensity, high-frequency, and long-duration; LHS: low-intensity, high-frequency, and short-duration; LLL: low-intensity, low-frequency, and long-duration; LLS: low-intensity, low-frequency, and short-duration

**Article title:** Effects of different combinations of mechanical stress intensity, duration, and frequency on the articular cartilage in mice

**Journal name:** Molecular Biology Reports

**Author names:** Yoshio Wakimoto, Yasushi Mimura, Shota Inoue, Masato Nomura, Hideki Moriyama

**Affiliation and e-mail address of the corresponding author:**

Department of Rehabilitation Science, Kobe University Graduate School of Health Sciences

wakio0820.kbu@gmail.com
